# Cultivation of Clear Cell Renal Cell Carcinoma Patient-Derived Organoids in an Air-Liquid Interface System as a Tool for Studying Individualized Therapy

**DOI:** 10.3389/fonc.2020.01775

**Published:** 2020-09-22

**Authors:** Laura K. Esser, Vittorio Branchi, Sonia Leonardelli, Natalie Pelusi, Adrian G. Simon, Niklas Klümper, Jörg Ellinger, Stefan Hauser, Maria A. Gonzalez-Carmona, Manuel Ritter, Glen Kristiansen, Hubert Schorle, Michael Hölzel, Marieta I. Toma

**Affiliations:** ^1^Institute of Pathology, University Hospital Bonn, Bonn, Germany; ^2^Department of General, Visceral, Thoracic and Vascular Surgery, University Hospital Bonn, Bonn, Germany; ^3^Medical Faculty, Institute of Experimental Oncology, University Hospital Bonn, Bonn, Germany; ^4^Department of Urology, University Hospital Bonn, Bonn, Germany; ^5^Department of Internal Medicine I, University Hospital Bonn, Bonn, Germany

**Keywords:** RCC, organoid, ccRCC, pRCC, targeted therapy, immunotherapy, ALI PDO

## Abstract

Clear cell renal cell carcinoma (ccRCC) is the most common renal cancer accounting for 80% of all renal cancers as well as the majority of renal cancer-associated deaths. During the last decade, the treatment paradigm for ccRCC has radically changed. In particular, the recent development of immune checkpoint inhibitors (ICI) has led to an increased overall survival in the metastatic setting. Moreover, novel immune therapies targeting the tumor microenvironment have been developed. In this rapidly evolving treatment landscape, precise tools for personalized cancer therapy are needed. Here, we collected fresh tissue from 42 patients who underwent surgical resection for renal cell carcinoma. Part of the tissue was used to obtain formalin-fixed, paraffin-embedded samples or RNA. The remaining tissue was minced and cultured in a collagen-based three-dimensional, air-liquid interface (ALI) culture system. The generated patient-derived tumor organoids (ALI PDOs) were characterized by immunohistochemistry staining and RNA sequencing to validate their close similarity to the matched tumor. Immune cells and stromal cells within the microenvironment could be identified. Finally, we treated 10 ALI PDOs with the commonly used targeted cancer drug cabozantinib or the ICI nivolumab. Interestingly, we observed varying responses of ALI PDOs to these treatments and future studies are needed to investigate whether the ALI PDO approach could inform about treatment responses in patients. In conclusion, this three-dimensional ccRCC culture model represents a promising, facile tool for monitoring tumor responses to different types of therapies in a controlled manner, yet, still preserves the key features of the tumor of origin.

## Introduction

Renal cancer affected about 403,300 people and led to approximately 175,000 deaths globally in 2018 (1). The most common form of renal cancer is renal cell carcinoma (RCC), which is a heterogeneous disease consisting of three histologically discriminable main subtypes, namely clear cell renal cell carcinoma (ccRCC), papillary RCC (pRCC), and chromophobe RCC (chRCC) (2). CcRCC is the most common subtype making up 80% of all cases (3). Due to high resistance against radiation and conventional chemotherapy, the treatment options for metastatic renal cancer were limited (4), but the options changed dramatically during the last years. Three clinical trials investigated the efficacy of novel immune targeting agents in treatment-naïve metastatic RCC and led to the approval of different combination therapies with immune checkpoint inhibitors (ICI) as the backbone, which became the new first-line standards (5–7). All evaluated combination therapies of ICI plus ICI or ICI plus tyrosine kinase inhibitors (TKI) improved the overall survival significantly and achieved remarkable objective response rates: 51.4% for avelumab plus axitinib (6), 42% for nivolumab plus ipilimumab (5), and 59.3% for pembrolizumab plus axitinib (7). Nevertheless, a large proportion of the patients had no clinical benefit indicating that the heterogeneity of RCC remains a clinical challenge. Moreover, combination therapies led to a high rate of side effects (8). Hence, the prediction of therapy responses needs intensive further research. The JAVELIN Renal 101 study using avelumab plus axitinib included 63.2% PD-L1 positive RCCs. The objective response rate of PD-L1 positive tumors was 55.2% compared to 51.4% in the overall cohort, which indicates that PD-L1 expression is not a reliable predictor for therapy response in RCC (9).

By establishing models, which resemble the patient’s situation more closely, many research groups hope that the adequate therapy for the individual patient can be determined with a higher probability. Over the past years, patient-derived organoids (PDOs) from many different tumor types have been established and gained interest as a tool for drug screening (10–12).

For renal cancer, only a few studies using patient-derived material such as primary cell lines or tumor (stem) cell-derived organoids have been published with regard to drug testing (13–15). Still, the absence of stromal and immune cells limit these approaches when considering appropriate tools for studying personalized cancer therapy including ICI targeting the PD-1/PD-L1 axis. Neal and colleagues developed PDOs in an air-liquid interface (ALI) system, which resemble not only the tumor histology but also the tumor immune microenvironment making ALI PDOs promising tools for personalized cancer therapy studies *in vitro* (16). Here, we used the protocol of Neal and colleagues to cultivate 42 air-liquid interface patient-derived organoids (ALI PDOs) from renal tumors, characterized them by different approaches and examined the treatment response to cabozantinib and nivolumab.

## Materials and Methods

### Human Tumor Specimens

Prior to tumor resection, the patients’ consent was obtained from the patients undergoing surgery. Human tumor samples were surgically resected at the University Hospital Bonn. The experiments were approved by the Ethics Committee of Bonn University Hospital (417/17 and 96/19). Tumor tissue was obtained from treatment-naïve patients, who underwent partial or radical nephrectomy between 2019 and 2020 in the Department of Urology, University Hospital Bonn. Pathological evaluation confirmed the malignancy of the samples.

### ALI PDO Culture

Tissues from resected tumors were cut thoroughly on ice, washed three times with ADMEM/F12 (Thermo Fisher) containing 1x Normocin (InvivoGen). Subsequently, the minced tissue pieces were resuspended in 1 ml Type I collagen solution containing 10x Ham‘s F12 (Ham’s F-12 Nutrient Mix powder, Thermo Scientific) and reconstitution buffer (2.2 *g* NaHCO3 in 100 ml, 0.05 N NaOH, 200 mM HEPES) in a ratio of 8:1:1, respectively. Next, the fragment-collagen solution was added on top of a 0.4 μm transwell insert (PICM03050, Millicell-CM, Millipore), which was previously coated with 1 ml of the mentioned collagen I solution containing 10x Ham’s F12 and reconstitution buffer. The transwell insert was placed into a regular 6-well and left to solidify for 30 min in a 37°C incubator. After solidification, 1 ml of ADMEM/F12 supplemented with 50% Wnt3a, R-spondin 1 conditioned medium with 1 mM HEPES (Thermo Fisher), 1x Glutamax (Thermo Fisher), 10 mM Nicotinamide (Sigma), 1 mM N-Acetylcysteine (Thermo Fisher), 1x B27 without vitamin A (Thermo Fisher), 0,5 μM A83-01 (Sigma), 1x Penicillin/Streptomycin (Thermo Fisher), 10 nM Gastrin (Sigma), 10 μM SB-202190 (Peprotech), 50 ng ml^–1^ EGF (Sigma), 25 ng ml^–1^ Noggin (Invitrogen), 100 μg ml^–1^ Normocin, and 600 units ml^–1^ IL-2 (Peprotech) was added. Passaging of ALI PDOs was performed by addition of 200 units ml^–1^ collagenase IV to the insert and incubation for 30 min at 37°C until the collagen was dissociated. Next, three washing steps with PBS and EDTA were conducted to inhibit the activity of the collagenase. ALI PDOs were taken up by 1 ml Type I collagen solution as described above and replated at desired mass density into new ALI collagen gels. Cryopreservation was performed by dissociating the collagen as described above, washing and resuspension in CryoStor CS10 (HemaCare).

### Haematoxylin and Eosin Staining

For haematoxylin and eosin (HE) stains the grown ALI PDOs within the collagen gels were cut out with a scalpel and fixed in formalin for 30 min. Subsequently, the fixed ALI PDOs were washed three times with PBS and HistoGel (Richard-Allan Scientific) was added according to the manufacturer’s protocol. The samples were left to cool down and solidify at 4°C and embedded in paraffin. HE staining was performed according to established staining protocols of our routine laboratory.

### Immunohistochemistry

For immunohistochemistry (IHC) staining, the ALI PDOs were processed as described above. IHC staining of the various antibodies was performed according to established staining protocols of the routine laboratory. The following antibodies were used: PAX8 (dilution 1:100, clone MRQ-50, Cell Marque), vimentin (dilution 1:5000, clone V9, Agilent), PD-L1 (dilution 1:50, clone E1L3N, Cell Signaling Technology), CA9 (dilution 1:8000, clone EPR 23055-5, Abcam), CD8 (dilution 1:50, clone C8/144B, Agilent), LCA (dilution 1:2000, clone 2B11 + PD7/26, Agilnet), and Granzyme B (dilution 1:50, clone 11F1, Leica). Stainings were performed on an Autostainer 480S (Fa Medac).

### RNA Sequencing

RNA from ALI PDOs and corresponding tissue was extracted using a Roboklon Kit. Raw single-read sequencing results were mapped to the human genome (GRCh38) with hisat2-2.1.0 (17). The mapped reads were processed using samtools (18) and featureCounts (19) to quantify reads. Read counts were statistically analyzed with the Bioconductor software package DESeq2 (20). Differentially expressed genes were calculated for each of the groups by applying multiple testing corrections including Bonferroni correction method and FDR. A FDR cutoff of 0.05 was accepted as significant. The PCA plot and Volcano plot were generated using ggplot2 and EnhancedVolcano (21, 22). The differentially expressed genes were further analyzed using the Hallmark gene set collection (Molecular Signature Database, Broad Institute). A pre-ranked list with differentially expressed genes was called into the Gene Set Enrichment Analysis (GSEA) software with default settings for hallmark gene sets (23).

### Therapy Testing

Air-liquid interface patient-derived organoids were cultivated for 1 week before starting the treatment. One insert each was treated with the targeted therapy cabozantinib (2.5 μM), the immunotherapy nivolumab (10 μg ml^–1^) or kept as a control. Media including the corresponding therapy was changed every 3 days. After 1 week, the ALI PDOs in the collagen gels were embedded as described above. The sections were stained with HE to visualize the degree of necrosis. The slides were digitalized with a Zeiss Mirax scanner and the area of viable cells in comparison to the total area was measured using Fiji (24). In case of no decrease in viability, no response was indicated (“–“), up to 1/3 reduction in viability was indicated as a weak response (“+”), up to 2/3 reduction as a medium response (“++”), and more than 2/3 reduction as a strong response (“+++”).

## Results

### Establishment of a Patient-Derived Kidney Tumor ALI PDO Biobank

**FIGURE 1:**
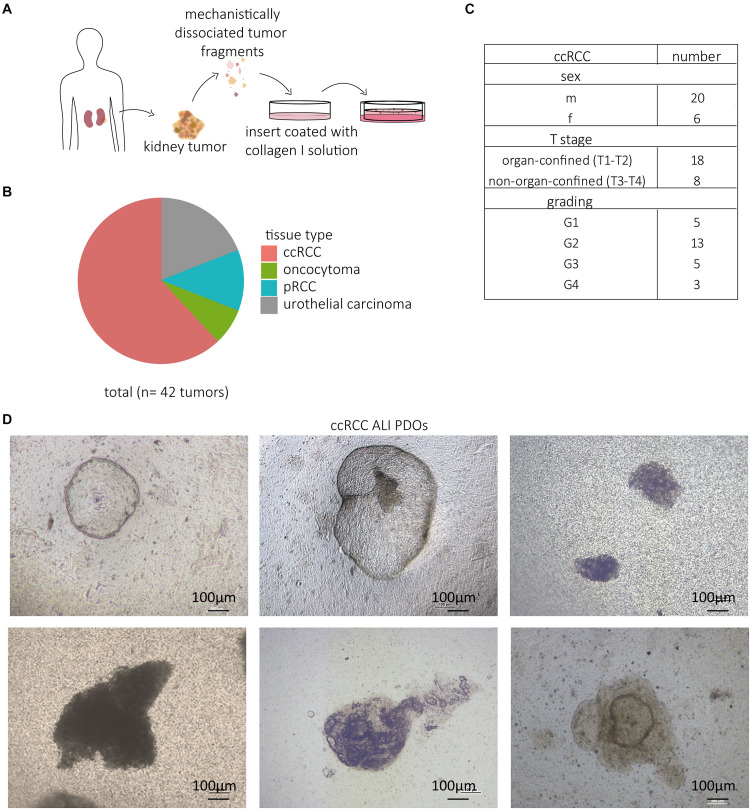
Established ccRCC ALI PDOs. Resected tumor tissue was cultivated by mechanistic fragmentation and subsequent uptake in a collagen I solution. Ultimately, the fragmented kidney tissue was plated in a collagen-coated insert and placed in a cell culture dish **(A)**. 42 kidney tissue types were taken into culture, namely 26 ccRCCs, 5 pRCCs, 8 urothelial carcinomas, and 3 oncocytomas **(B)**. Clinical data, including sex, T stage and grading, of the cultured ccRCC ALI PDOs **(C)**. Light microscopy pictures of cultivated kidney PDOs taken at 5x magnification **(D)**.

Neal and colleagues established a protocol to cultivate PDOs in an ALI system, which sustains the complex structure of the tissue of origin (16). The obtained tissue was fragmented into small pieces and plated within a collagen I matrix on top of a coated insert. Based on this protocol, we established 42 ALI PDOs from different renal tumors, which were surgically resected (Figures 1A,B). Our study included the most common subtypes of RCC, namely ccRCC, and papillary carcinoma (pRCC). In addition, we successfully cultivated upper urinary tract urothelial carcinomas, which at times occur in the renal pelvis. From the 42 samples, 26 were confirmed as ccRCC (Figure 1B and Supplementary Table 1). In 77% (20/26) of the cases, we successfully established ALI PDOs, which could be passaged and remained viable for more than 30 days in culture. The tissue for the cultivated ALI PDOs was obtained from 20 male and six female patients ranging in age from 33 to 87 years. 18 tumors were organ-confined (T1–T2) and eight non-organ-confined (T3–T4). The majority of the tumors was graded as G2 (13 tumors), while the remaining tumors were evenly distributed between G1, G3, and G4 (five tumors in G1, five tumors in G3, and three tumors in G4; Figure 1C).

The success rate for pRCC was 80% (4/5) and for urothelial carcinoma 88% (7/8). In addition, we effectively established ALI PDOs from one oncocytoma (1/3), a benign renal tumor. Yet, we failed to cultivate the single case of the rare RCC subtype chromophobe renal cell carcinoma (0/1). Overall, cultivation of the ALI PDOs was successful in 72%.

The ALI PDOs appeared phenotypically as roundish, dense structures (Figure 1D), only a few cases showed cystic organoid structures.

The expansion rates varied between samples. In general, ALI PDOs could be passaged after 21–30 days with 1:3 split ratios. It was possible to cryopreserve and recover the ALI PDOs as described (16).

We next sought to further characterize the ccRCC ALI PDOs by histological staining and RNA sequencing (RNA Seq) to validate the ALI PDOs as a suitable platform for studying personalized cancer therapy.

### Kidney Tumor ALI PDOs Resemble Tumor of Origin Histologically

**FIGURE 2:**
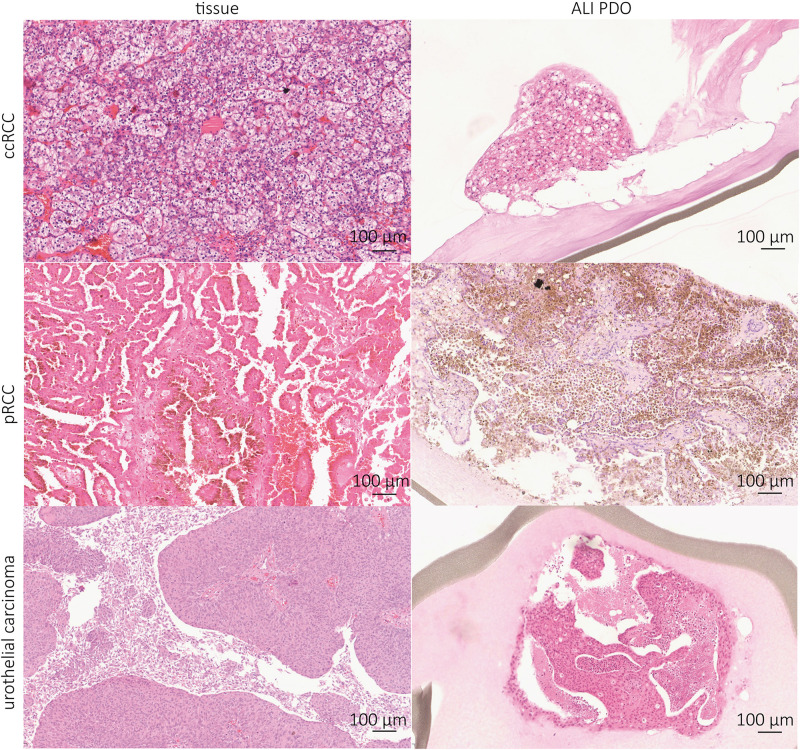
Kidney tumor ALI PDOs resemble tumor of origin histologically. IHC stainings were prepared to compare the generated ALI PDOs with their derived tissue. ccRCC, pRCC, and urothelial carcinoma show the same tissue structure as their corresponding tissues. Pictures were taken at 10x magnification.

To verify the similarity between the tumor of origin and our cultivated ALI PDOs, IHC stainings were performed. HE staining revealed that the tumor histology resembled the complex histological structure of the tissue of origin. The growth pattern of the ALI PDOs was solid in most cases (Figure 2). In two cases, the ALI PDOs showed a cystic phenotype.

**FIGURE 3:**
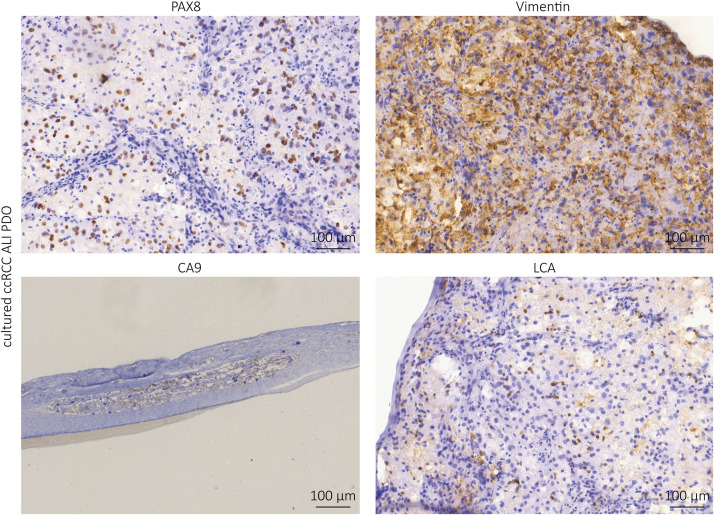
Immunhistochemistry staining of the cultured ccRCC ALI PDOs. The ALI PDOs show positivity for PAX8, Vimentin, CA9, and LCA.

Further IHC analyses showed positivity for PAX8, a marker of renal epithelial origin; CA9, a characteristic marker of ccRCC; vimentin, a marker for stromal cells and LCA (lymphocyte common antigen), a marker of lymphocytes (Figure 3). Our results indicate that the complex tissue architecture, phenotype and cellular composition of the primary tumors were maintained.

### Immune Cells and Expression of PD-L1 Immune Checkpoint Proteins

**FIGURE 4:**
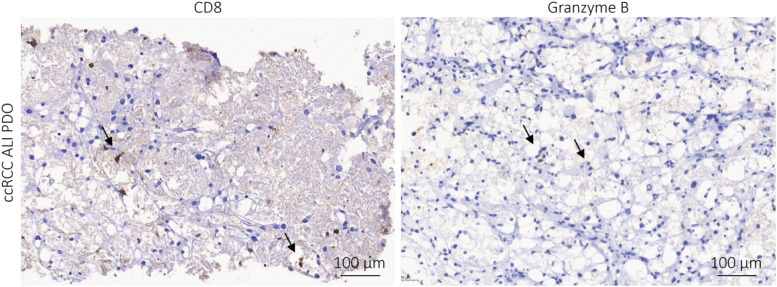
Immune cells are preserved in the ALI PDOs. ALI PDOs were stained for CD8 and granzyme B to validate the presence of immune cells. Asterisks indicate positive stained cells. Pictures were taken at 20x magnification.

Current treatment options for ccRCC are surgery and targeted therapy (TKI), as well as ICI (5–7). Thus, we checked for the presence of the immune checkpoint proteins PD-1 and PD-L1 in the primary tissues. Whereas the expression of PD-1 ranged from 0 to 80% on the immune cells, the expression of PD-L1 was much lower on the tumor cells ranging from 0 to 5%. The presence of cytotoxic T cells, which are CD8-positive, varied substantially. While some tissues contained more than 100 CD8^+^ cells per high power field (HPF), others showed less than ten CD8^+^ T cells per HPF. Individual cells stained positive for the cytotoxic immune cell marker granzyme B, which could also be NK cells besides CD8^+^ T cells (Table 1). IHC staining of ALI PDOs showed positivity for CD8 and granzyme B (Figure 4). Thus, the persistence of cytotoxic immune cells in ALI PDOs suggested that ALI PDOs were suitable models to test the efficacy of ICI targeting the PD-1/PD-L1 axis.

**TABLE 1:** Tumor tissue was stained and examined for CD8, granzyme B, PD-1, and PD-L1 positivity to draw conclusions on therapy response.

ALI PDO	CD8	Granzyme B	PD-1	PD-L1
1	80/HPF	Negative	5% Immune cells	Negative
2	35/HPF	Individual cells	3% Immune cells	Negative
3	25/HPF	Negative	Negative	Negative
4	20/HPF	Negative	Negative	Negative
5	90/HPF	Individual cells	5% Immune cells	1% Immune cells
6	70/HPF	Individual cells	80% Immune cells	Negative
7	>100/HPF	Negative	20% Immune cells	5% Tumor cells
8	2/HPF	Negative	Negative	Negative

### RNA Sequencing Demonstrates Close Molecular Similarity of ALI PDOs and Tissue of Origin

**FIGURE 5:**
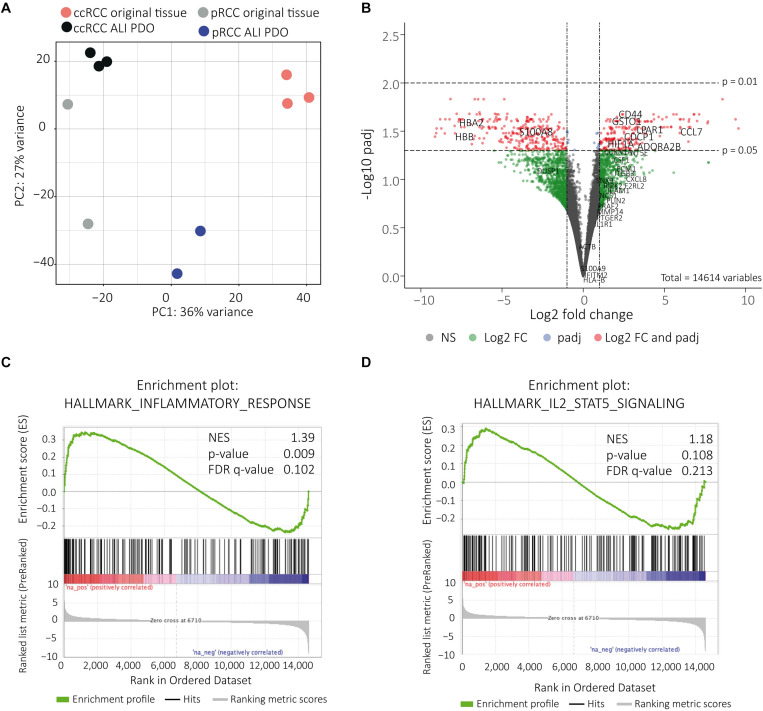
RNA sequencing shows close molecular relationship. The close molecular relationship of the samples’ gene expression profiles was projected onto the first two principal components in a PCA plot **(A)**. Volcano plot displaying the differentially expressed genes. Genes of interest, which are associated with inflammatory response and IL-2 STAT 5 signaling, are indicated in the up-regulated genes, genes expressed in blood are indicated in down regulated genes **(B)**. Genes correlated with inflammatory response were up-regulated. NES = normalized enrichment score **(C)**. Genes correlated with IL-2 STAT5 signaling were up-regulated. NES = normalized enrichment score **(D)**.

Total RNA isolated from ALI PDOs was analyzed by 3′ mRNA-Seq and compared to the matched tumors. Only 418 genes(FDR < 0.05, | log2FC (2)| were differentially expressed. Among those 161 were up-regulated and 257 down-regulated. Using principal component analysis (PCA) we were able to visualize the close relationship of the samples’ gene expression profiles. The samples belonging to the ALI PDOs and the tumors of origin cluster together, respectively. In addition, we also observed that ccRCC and pRCC subtypes were closer to each other (Figure 5A). Differentially expressed genes were also illustrated in a volcano plot (Figure 5B). Of note, some of the strongly down-regulated genes in ALI-PDOs are known to be highly expressed in blood cells such as hemoglobin genes. Obviously, this difference is explained by the lack of blood circulation of ALI PDOs. Subtracting this expected gene difference further supported the molecular similarity between ALI PDOs and matched tumors of origin. Using the Hallmark gene set collection (Molecular Signature Database, Broad Institute), GSEA showed an up-regulation for 5 gene sets (FDR < 0.05; Supplementary Table 2) and down-regulation of 4 gene sets (FDR < 0.05; Supplementary Table 3). Among the top 20 up-regulated gene sets were also the gene sets inflammatory response (Figure 5C) and IL-2 STAT5 pathway (Figure 5D and Supplementary Tables 2, 3). However, the FDR for the enrichment of the inflammatory response was 0.102 and for the IL-2 STAT5 pathway 0.213. In addition, the normalized enrichment scores (NES) were relatively moderate with 1.39 and 1.18 respectively (Figures 5C,D). These up-regulated genes involved in inflammatory response and IL-2 STAT5 pathway were highlighted in the volcano plot (Figure 5B).

### Treatment Shows Different Response Rates for Individual ALI PDOs

**FIGURE 6:**
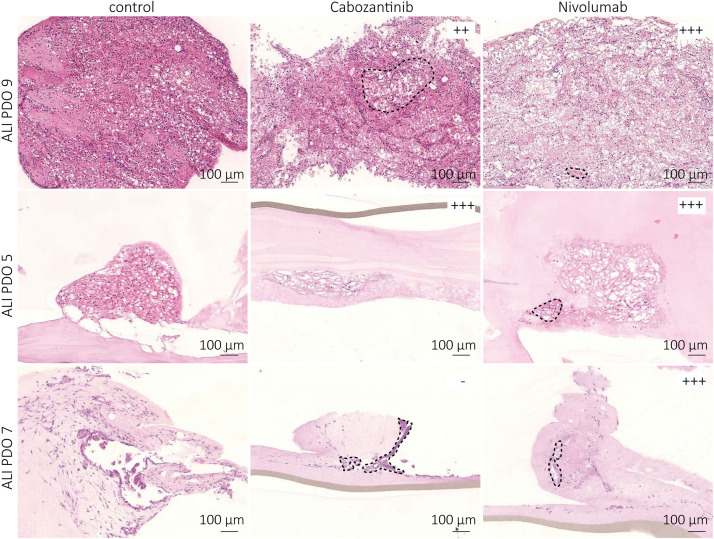
Treated ALI PDOs show drastically different responses. ALI PDOs were treated with either 2.5 μM cabozantinib or with 10 μg ml^– 1^ nivolumab for 1 week. ALI PDOs were embedded and HE stainings prepared. ALI PDO 9 showed a stronger response toward nivolumab, ALI PDO 5 showed a similar response toward cabozantinib and nivolumab, ALI PDO 7 responded only to nivolumab. Dashed lines indicate areas of viable cells in treated ALI PDOs.

Histological and molecular resemblance of the generated ALI PDOs compared to the matched tumors suggested their suitability for therapy testing. Therefore, we treated ten of the ALI PDOs either with the TKI cabozantinib (2.5 μM) or with the anti-PD-1 ICI nivolumab (10 μg ml^–1^) for 1 week. After termination of the experiment, we analyzed the treated patient ALI PDOs for viable cell areas compared to the control group and observed differences in response. The control group showed no substantial decrease in viability. Whereas some PDOs responded to both cabozantinib and nivolumab resulting in a similar degree of necrosis (ALI PDO 1, 5, 6), others showed solely a response to nivolumab (ALI PDO 7), or no response at all (ALI PDO 10; Figure 6). Most ALI PDOs responded better to one of the therapies (Table 2). Interestingly, the response of the ALI PDOs toward nivolumab appeared to depend on the amount of CD8^+^ cells in the matched tumor tissue. ALI PDOs, which responded well to nivolumab had more than 35 CD8^+^ cells per HPF in its corresponding tissue (Table 1).

**TABLE 2:** The response rate of the ALI PDOs was determined by measuring the area of viable cells in comparison to the whole area with Fiji.

ALI PDO	Treatment	Response
	Control	–
1	Cabozantinib	+
	Nivolumab	++
	Control	–
2	Cabozantinib	++
	Nivolumab	+++
	Control	–
3	Cabozantinib	++
	Nivolumab	+++
	Control	–
4	Cabozantinib	+++
	Nivolumab	++
	Control	–
5	Cabozantinib	++
	Nivolumab	++
	Control	–
6	Cabozantinib	++
	Nivolumab	++
	Control	–
7	Cabozantinib	–
	Nivolumab	+++
	Control	–
8	Cabozantinib	++
	Nivolumab	+++
	Control	–
9	Cabozantinib	++
	Nivolumab	+++
	Control	–
10	Cabozantinib	–
	Nivolumab	–

*“−“: no response, “+”: weak response (up to 1/3 reduction in viablility), “++”: medium response (up to 2/3 reduction in viability), and “+++”: strong response more than (2/3 reduction in viability; B).*

In summary, ALI PDOs responded to different types of clinical treatments for ccRCC, cabozantinib and nivolumab, respectively.

## Discussion

The substantial heterogeneity within RCC demands for personalized treatment strategies. Despite the increase in treatment options in the last years, the patient’s response remains poorly predictable (5–7). To determine the best therapy, new model systems, which resemble the patient’s situation as closely as possible, are required. As of now, drug screening patient-specific models for RCC are scarce (13–15).

Therefore, we tested patient-derived ALI PDOs, which were generated using an ALI system as previously described (16). We were successful in cultivating 72% (31/43) of all cases and 77% (20/26) of ccRCCs, which is in agreement with the results of Neal and colleagues (16).

We confirmed the close relationship of the ALI PDOs and the tumor tissue of origin by IHC staining and RNA sequencing. Not only is the complex structural histology preserved (Figure 2), but also immune and stromal cells (Figures 3, 4). The PCA Plot shows the close correlation between the ALI PDOs and their corresponding tissue. Whereas all ccRCC and pRCC ALI PDOs are clustering in close proximity to their corresponding tissue, the clustering of the different RCC subtypes differs from each other. This additionally highlights the minor differences between the ALI PDOs and the matched tumor. Differential expression analysis resulted in only 418 differently expressed genes (FDR < 0.05). Among the most down regulated genes were genes highly expressed in whole blood including hemoglobins (HBB and HBA1), which was expected and explained by the lack of blood circulation in ALI PDOs (Figure 5B). Altogether, we concluded that ALI PDOs and matched tumor of origin differ only by a moderate number of differentially expressed genes supporting their close molecular similarity. This is also supported by GSEA, which showed moderate enrichment of gene sets associated with inflammatory responses such as IL-2 STAT5 signaling. This again, underlines the similarity of the ALI PDOs to the matched tumors. The results of GSEA are likely explained by the fact that the culture medium contains IL-2 in order to maintain infiltrating lymphocytes in ALI PDOs (16), which may result in the release of pro-inflammatory cytokines. In the future, the right amount of stimulation by IL-2 and preservation of the original tumor environment needs to be carefully determined, in particular when optimizing this platform toward prediction of responses to ICI.

The small differences between ALI PDOs and the tumor tissues make it possible to focus on the immune responses within the tumor, including resident immune cells without recruitment from the periphery. Nevertheless, it is not restricted to the intratumoral immune response only. In recent years, adoptive cell therapy has gained interest and added additional therapies for patients (25). Co-culturing of ALI PDOs with tumor infiltrating lymphocytes (TIL), gene-modified T cells expressing novel T cell receptors (TCR), or chimeric antigen receptors (CAR) may provide additional information in this promising field and for possible treatment options.

To date, generated kidney organoids consisted mostly of epithelial structures lacking stromal and immune cells (14, 15, 26). Some of these models have proven to be suitable for development of personalized treatment. For example, Fendler et al. generated ccRCC stem cell based organoids and spheroids to test WNT and Notch inhibition as a potential new therapy. However, ALI PDOs provide the additional benefit that therapeutic effects can also be addressed on the tumor microenvironment. This is of pivotal importance for immunotherapy, including ICI.

Renal cell carcinoma is considered an immunogenic cancer due to its high immune infiltration (27). Thus, ICI blocking PD-1/PD-L1 became standard of cares in treatment-naïve and pre-treated patients with ccRCC (5, 6). Nevertheless, novel therapies and combinations are needed. The presence of stromal and immune cells in ALI PDOs indicated the applicability, not only for targeted therapy, but also for immunotherapy testing. In concordance with the clinical situation, the treated ALI PDOs showed different responses to targeted therapy and immunotherapy. The observed dependency on CD8^+^ cells of nivolumab treatment is in line with recent publications, which revealed the importance of CD8^+^ TIL in therapy response on ICI in several tumors such as melanoma (28) and renal carcinoma (29).

In addition, the immune cells showed PD-1 expression, albeit to very different amounts (ranging from 3% to 80%; Table 1). However, in some cases PD-1 was hardly detected by IHC, but still, nivolumab treatment resulted in a decrease in viability (Tables 1, 2). The tissue of which ALI PDO 10 was derived from, lacked PD-1/PD-L1 and Granzyme B expression and had only 2 CD8^+^ cells per HPF (Table 1). It responded neither to cabozantinib nor to nivolumab supporting the dependency of nivolumab responses on CD8^+^ T cell infiltrations. Additionally, no conclusions in terms of response rates from PD-L1 expression in the tissue of origin could be drawn, which is in agreement with literature stating that PD-L1 is not a reliable marker for the prediction of therapy response (9).

In conclusion, the generated ALI PDOs are a suitable tool for therapy testing. Additionally, new therapies can be tested for its efficacy. We believe that it is beneficial to start treating the ALI PDOs at an early time point to minimize the accumulation of genetic differences due to potential culture pressure, but this needs to be carefully determined. This guarantees the close comparability between the tumor ALI PDOs and the patient’s tumor. Yet, future prospective studies need to be conducted to draw conclusions for the correlation between the therapy response in the ALI PDOs and the corresponding patient demonstrating its usability as a preclinical tool.

## Data Availability Statement

The datasets presented in this study can be found in online repositories. The names of the repository/repositories and accession number(s) can be found below: Sequence Read Archive (SRA) (https://www.ncbi.nlm.nih.gov/, PRJNA634836).

## Ethics Statement

The studies involving human participants were reviewed and approved by Ethics Committee of Bonn University Hospital (417/17 and 96/19). The patients/participants provided their written informed consent to participate in this study.

## Author Contributions

MT and MH designed the experiments and supervised the progress throughout the study. LE and VB carried out the experiments. SL, AS, and NK helped drafting the manuscript and interpreting the data. HS, NP, and GK helped designing the experiments. JE, MR, SH, and MG-C planned, critically revised the manuscript, and supported the clinical patient data. LE and MH analyzed the sequencing data. MT, MH, and LE wrote the manuscript. All authors contributed to the article and approved the submitted version.

## Conflict of Interest

The authors declare that the research was conducted in the absence of any commercial or financial relationships that could be construed as a potential conflict of interest.
