# Supplementary material for: Cultivation of Clear Cell Renal Cell Carcinoma Patient-Derived Organoids in an Air-Liquid Interface System as a Tool for Studying Individualized Therapy
Source: Front Oncol. 2020 Sep 22;10:1775. doi: 10.3389/fonc.2020.01775 (PMC7537764; doi:10.3389/fonc.2020.01775)

Supplementary Material

# Supplementary Data

**Supplementary Table 1.** Clinical data of all cultured ALI PDOs from different tumor types, namely ccRCC, pRCC, oncocytoma and urothelial carcinoma.

| **ccRCC** | **Number** |
| --- | --- |
| **Age** |  |
| Range | 33-87 |
| Mean | 68.53 |
| Median | 69 |
| **Sex** |  |
| m | 20 |
| f | 6 |
| **T Stage** |  |
| organ-confined (T1-T2) | 18 |
| non-organ-confined (T3-T4) | 8 |
| **Grading** |  |
| G1 | 5 |
| G2 | 13 |
| G3 | 5 |
| G4 | 3 |
| **pRCC** | **Number** |
| **Age** |  |
| Range | 61-84 |
| Mean | 69.6 |
| Median | 67 |
| **Sex** |  |
| m | 4 |
| f | 1 |
| **T Stage** |  |
| organ-confined (T1-T2) | 4 |
| non-organ-confined (T3-T4) | 1 |
| **Grading** |  |
| G1 | 2 |
| G2 | 3 |
| G3 | - |
| G4 | - |
| **oncocytoma** | **Number** |
| **Age** |  |
| Range | 65-68 |
| Mean | 66 |
| Median | 65 |
| **Sex** |  |
| m | 2 |
| f | 1 |
| **T Stage** |  |
| organ-confined (T1-T2) | - |
| non-organ-confined (T3-T4) | - |
| **Grading** |  |
| G1 | - |
| G2 | - |
| G3 | - |
| G4 | - |
| **urothelial carcinoma** | **Number** |
| **Age** |  |
| Range | 46-86 |
| Mean | 68.75 |
| Median | 70 |
| **Sex** |  |
| m | 5 |
| f | 3 |
| **T Stage** |  |
| organ-confined (T1-T2) | 2 |
| non-organ-confined (T3-T4) | 6 |
| **Grading** |  |
| low grade | - |
| high grade | 8 |
|  |  |

**Supplementary Table 2.** Top 20 upregulated hallmark gene sets for differentially expressed genes.

| Hallmark | NES | raw p-value | FDR q-value | FWER p-  value |
| --- | --- | --- | --- | --- |
| Allograft rejection | 2.365 | 0.000 | 0.000 | 0.000 |
| MTORC1 signaling | 1.838 | 0.000 | 0.012 | 0.012 |
| Reactive oxygen species pathway | 1.753 | 0.003 | 0.012 | 0.020 |
| MYC targets V1 | 1.632 | 0.000 | 0.030 | 0.061 |
| Complement | 1.600 | 0.000 | 0.033 | 0.082 |
| Epithelial mesenchymal | 1.516 | 0.000 | 0.065 | 0.179 |
| Coagulation | 1.438 | 0.025 | 0.091 | 0.274 |
| Cholesterol homeostasis | 1.395 | 0.033 | 0.111 | 0.353 |
| Inflammatory response | 1.389 | 0.009 | 0.102 | 0.367 |
| KRAS signaling up | 1.322 | 0.009 | 0.146 | 0.515 |
| Apoptosis | 1.309 | 0.019 | 0.143 | 0.542 |
| Unfolded protein response | 1.279 | 0.054 | 0.161 | 0.623 |
| Protein secretion | 1.278 | 0.089 | 0.149 | 0.627 |
| Interferon alpha response | 1.250 | 0.091 | 0.170 | 0.702 |
| Spermatogenesis | 1.236 | 0.131 | 0.177 | 0.740 |
| Interferon gamma respone | 1.227 | 0.054 | 0.176 | 0.763 |
| DNA repair | 1.225 | 0.066 | 0.168 | 0.768 |
| IL-2 STAT5 signaling | 1.183 | 0.108 | 0.213 | 0.864 |
| IL6 JAK STAT3 signaling | 1.132 | 0.226 | 0.276 | 0.933 |
| E2F targets | 1.091 | 0.241 | 0.339 | 0.966 |

**Supplementary Table 3.** Top 20 down regulated hallmark gene sets for differentially expressed genes.

| Hallmark | NES | NOM p-value | FDR q-value | FWER p-value |
| --- | --- | --- | --- | --- |
| KRAS signaling down | -1.802 | 0.000 | 0.009 | 0.014 |
| WNT beta catenin signaling | -1.782 | 0.000 | 0.006 | 0.020 |
| pancreas beta cells | -1.699 | 0.012 | 0.010 | 0.046 |
| Hedgehog signaling | -1.619 | 0.014 | 0.021 | 0.126 |
| Estrogen response late | -1.510 | 0.005 | 0.060 | 0.370 |
| Estrogen response early | -1.439 | 0.011 | 0.098 | 0.594 |
| TGF beta signaling | -1.403 | 0.055 | 0.122 | 0.738 |
| Angiogensis | -1.395 | 0.081 | 0.115 | 0.769 |
| Notch signaling | -1.332 | 0.123 | 0.171 | 0.919 |
| Hypoxia | -1.278 | 0.076 | 0.235 | 0.975 |
| TNFA signaling via NFKB | -1.212 | 0.115 | 0.346 | 0.998 |
| Mitotic spindle | -1.186 | 0.155 | 0.378 | 0.999 |
| Fatty acid metabolism | -1.166 | 0.197 | 0.391 | 1.000 |
| UV response up | -1.165 | 0.184 | 0.365 | 1.000 |
| Apical junction | -1.151 | 0.201 | 0.371 | 1.000 |
| Xenobiotic metabolism | -1.149 | 0.205 | 0.352 | 1.000 |
| Bile acid metabolism | -1.120 | 0.276 | 0.386 | 1.000 |
| Heme metabolism | -1.116 | 0.251 | 0.374 | 1.000 |
| Peroxisome | -1.095 | 0.305 | 0.398 | 1.000 |
| UV response down | -0.994 | 0.468 | 0.598 | 1.000 |


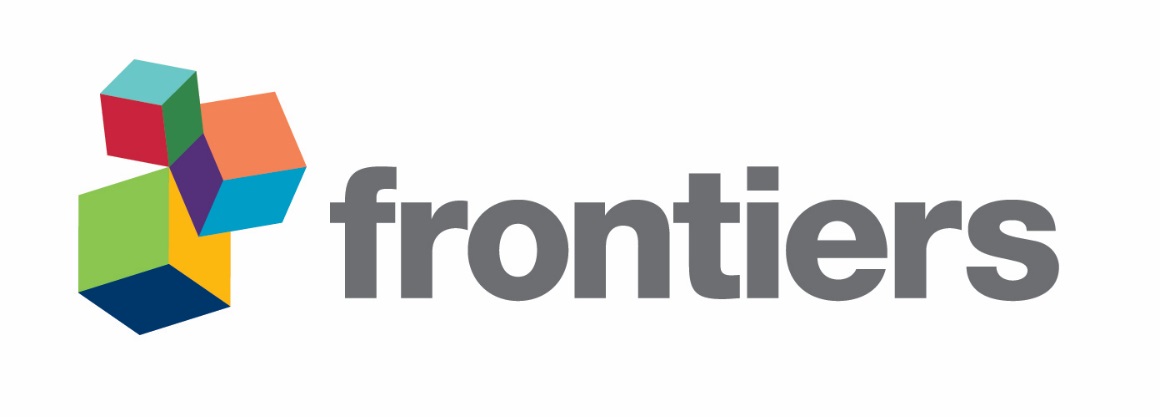

Supplement: Supplementary file 1 [file Data_Sheet_1.docx]
